# Decoding the ERD/ERS: influence of afferent input induced by a leg assistive robot

**DOI:** 10.3389/fnsys.2014.00085

**Published:** 2014-05-14

**Authors:** Giuseppe Lisi, Tomoyuki Noda, Jun Morimoto

**Affiliations:** ^1^ATR Computational Neuroscience Laboratories, Department of Brain Robot Interface, ATRKyoto, Japan; ^2^Mathematical Informatics Laboratory, Department of Information Science, Nara Institute of Science and TechnologyNara, Japan

**Keywords:** brain robot interface, assistive exoskeleton robot, ERD/ERS of the sensorimotor hand area, lower limb afferent input, ICA/CSP, wavelet, PCA/LDA, sparse logistic regression

## Abstract

This paper investigates the influence of the leg afferent input, induced by a leg assistive robot, on the decoding performance of a BMI system. Specifically, it focuses on a decoder based on the event-related (de)synchronization (ERD/ERS) of the sensorimotor area. The EEG experiment, performed with healthy subjects, is structured as a 3 × 2 factorial design, consisting of two factors: “finger tapping task” and “leg condition.” The former is divided into three levels (BMI classes), being left hand finger tapping, right hand finger tapping and no movement (Idle); while the latter is composed by two levels: leg perturbed (*Pert*) and leg not perturbed (*NoPert*). Specifically, the subjects' leg was periodically perturbed by an assistive robot in 5 out of 10 sessions of the experiment and not moved in the remaining sessions. The aim of this study is to verify that the decoding performance of the finger tapping task is comparable between the two conditions *NoPert* and *Pert*. Accordingly, a classifier is trained to output the class of the finger tapping, given as input the features associated with the ERD/ERS. Individually for each subject, the decoding performance is statistically compared between the *NoPert* and *Pert* conditions. Results show that the decoding performance is notably above chance, for all the subjects, under both conditions. Moreover, the statistical comparison do not highlight a significant difference between *NoPert* and *Pert* in any subject, which is confirmed by feature visualization.

## 1. Introduction

Brain-machine interfaces (BMI) have recently raised much interest in that they can help improve the quality of life of people with severe motor disabilities. In previous studies, the brain signal has been used to control devices that affect the surrounding environment such as wheelchairs or neuroprosthesis (Mussa-Ivaldi, 2000; Daly and Wolpaw, 2008; Pfurtscheller and Solis-Escalante, 2008). Regarding the nature of ERD/ERS, movement or preparation for movement is typically accompanied by a decrease in mu and beta rhythms, particularly contralateral to the movement (Wolpaw et al., 2002). This phenomenon has been named event-related desynchronization or ERD (Babiloni et al., 1999). Its opposite, rhythm increase, or event-related synchronization (ERS), occurs after movement and with relaxation (Pfurtscheller and Lopes da Silva, 1999). To note that similar ERD/ERS patterns in the sensorimotor area can be elicited also by motor imagery (Pfurtscheller and Neuper, 1997; Pfurtscheller and Lopes da Silva, 1999; McFarland et al., 2000). These features make it possible to use a BMI system based on ERD/ERS for the control of an external device (Pfurtscheller et al., 2000b; Wolpaw et al., 2003; Babiloni et al., 2007). If this is done irrespectively of any system cues, the control paradigm is called asynchronous and allows the subject to make self-paced decisions (Müller-Putz et al., 2006; Zhao et al., 2009; Solis-Escalante et al., 2010).

**Figure 1:**
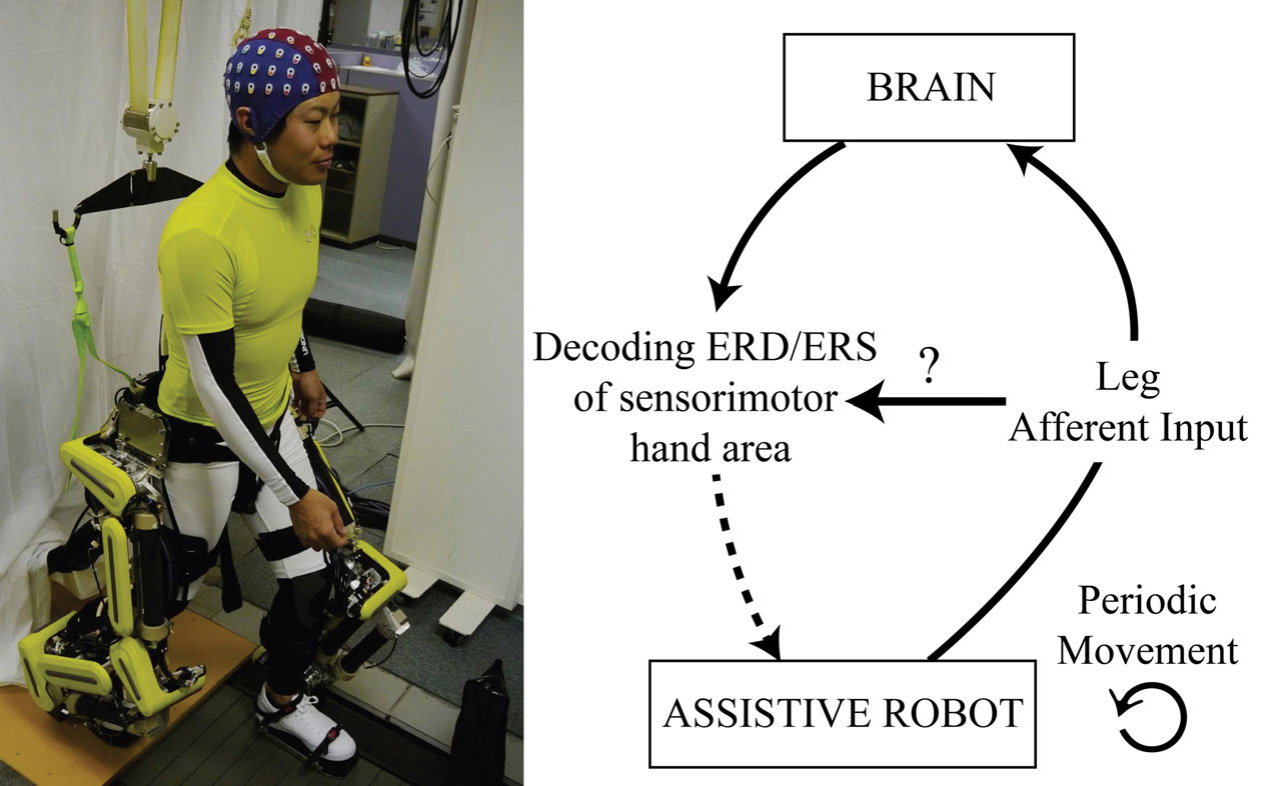
**Conceptual representation of the problem**. Understanding how the leg afferent input, induced by a leg assistive robot, influences the decoding of the ERD/ERS of the sensorimotor hand area. The paper focuses on the concepts represented by the solid arrows of this figure.

A similar control paradigm has yet to be carefully investigated for a lower limb exoskeleton robot. Of particular interest would be a BMI system, based on the ERD/ERS of the sensorimotor area, able to control an assistive robot (downward arrow of Figure 1). Similarly, Tsui et al. (2011); Huang et al. (2012) have succeeded in controlling a wheelchair by means of a BMI based on event-related (de)synchronization (ERD/ERS). Nonetheless, one of the differences between a wheelchair and a lower limb assistive robot is that the latter induces movements of the legs. In this context, the ERD/ERS phenomenon is not only related to active movements or motor imagery, but also to passive movements (upward arrow of Figure 1; Cassim et al., 2001; Müller et al., 2003; Müller-Putz et al., 2007; Wagner et al., 2012). Especially, Müller-Putz et al. (2007) highlighted that passive movements of the feet produce a significant ERD/ERS not only at the vertex, but over the whole sensorimotor cortex. This raises the question whether the somatosensory afferent input, induced by the periodic leg perturbation, interferes with the decoding ability of a BMI system based on the ERD/ERS of the sensorimotor hand area (horizontal arrow of Figure 1).

Specifically, in this paper, we investigate whether the periodic perturbation of lower limbs produces a significant decrease in the classification performance of actual right and left hand finger movements. The reason for decoding the right and left sensorimotor hand area stands in the reliability of the contralateral ERD/ERS spatial distribution (Wang et al., 2007) and in the fact that this approach is exhaustively discussed in literature (Guger et al., 2000; Pfurtscheller et al., 2000b; Blankertz et al., 2008a). Moreover, the *Idle* class is considered in order to simulate the behavior of the system in an online asynchronous setup (Müller-Putz et al., 2006).

Furthermore, the motivation for using real movements instead of motor imagery, is that the latter cannot be mechanically measured nor visually assessed by the experiment operator. As such, in case of misclassification, there is no certainty about whether the subject failed in generating a significant ERD/ERS by motor imagery or if the somatosensory afferent input of the leg actually affected the decoding. On the other hand, for real upper limb movements, the motor output is visible; therefore, we are certain that the motor cortex has produced a control command.

To note that even though patterns of mu and beta de/synchronization associated with actual movements are similar to those with motor imagery, they differ in magnitude (McFarland et al., 2000). Specifically, the spectral perturbations associated with motor imagery are considerably smaller than the ones of actual movements (Solis-Escalante et al., 2010). Nonetheless, according to Pfurtscheller et al. (1998), the beta ERS is significantly larger with hand as compared to finger movement. Therefore, the latter is chosen in this experiment, so as to deal with a spectral perturbation that is as close as possible to the one associated with motor imagery, while preserving the necessary property of objective measurability by the experiment operator.

Previous studies have dealt with EEG-based neuroprosthesis control (Pfurtscheller et al., 2000a; Müller-Putz et al., 2005, 2006, 2009), but to our knowledge this is the first time that the possibility of interference between the rhythms associated with passive and active movements is investigated.

## 2. Methods

Five healthy subjects were asked to perform real right/left-hand brisk finger tapping, or not to move (idle), while their brain activity was recorded by EEG. Each task lasted 2 s and was instructed by visual cues, interleaved by periods of rest of 8 s. Simultaneously, the subjects' leg was *perturbed by a periodic swing movement of a lower limb assistive robot* in 5 out of 10 sessions of the experiment. In this way, the experiment is characterized by two conditions: leg perturbed (*Pert*) and leg not perturbed (*NoPert*).

Therefore, the main question regarding the influence of the leg afferent input on the decoding of the sensorimotor area ERD/ERS can be stated as follows: *is the performance of finger tapping decoding significantly different between the *NoPert* and *Pert* conditions, for one or more subjects?* To answer this question, a classifier is trained to output the class of the finger tapping (*Idle, Left, Right*), given as input the features associated with the ERD/ERS. Cross-validated Kappa score (see section 2.3) is used to assess the decoding performance separately for *NoPert* and *Pert*. Then, individually for each subject, the Kappa scores of the two conditions are statistically compared by the *Z*-test (see Appendix). Moreover, it is important to perform an analysis that is as independent as possible from the characteristics of a specific decoder. For this reason, two types of feature extraction methods are used separately: *Unsupervised feature extraction* (ICA, Wavelet and PCA) and *Supervised feature extraction* (CSP, Wavelet, LDA).

As discussed in the introduction, actual left/right hand finger tapping and idle were chosen because of the following reasons:

1. the contralateral ERD/ERS spatial distribution associated with left or right hand movements can be reliably decoded;
2. actual movements are observable by the experiment operator, or mechanically measurable, therefore we are certain that an ERD/ERS activation must have been elicited by the motor task (with motor imagery the subject may fail to produce ERD/ERS);
3. finger movements are characterized by a significantly smaller ERS as compared to hand movements (Pfurtscheller et al., 1998), which makes the task as close as possible to a motor imagery one, while preserving the properties at point 1 and 2;
4. the idle class is used to simulate an online asynchronous setup.

### 2.1. Experimental setup

#### 2.1.1 Assistive robot specifications

**Figure 2:**
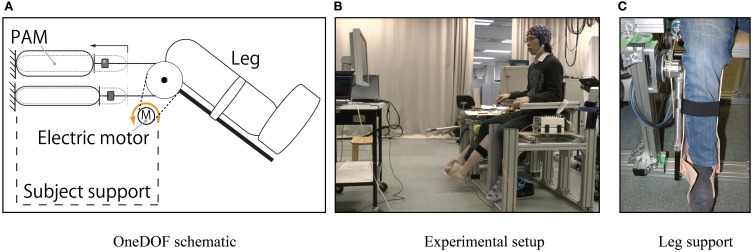
**Robot used during the experiment**. **(A)** PAM and electric actuators can exert parallel torques to move the leg. **(B)** Representation of the experimental setup. **(C)** Representation of the thermoplastic polymer leg support anchored to the robot and holding up the subject's leg.

Our custom made one degree of freedom robot (oneDOF, Noda et al., 2012) was used as assistive robot (Figure 2A). OneDOF is actuated using a pneumatic-electric hybrid strategy. In detail, two antagonistic pneumatic artificial muscles (PAM) generate large force by converting pressured gas energy into contraction force through their rubber tube. The advantage of using a PAM is that it can exert very large torques (maximal 70 Nm), while generating insignificant electromagnetic noise from the point of view of the EEG system. Moreover, an electric motor generates parallel small torque (maximal 5 Nm) in order to make fast and precise corrections to the torque generated by PAM. This strategy allows for both a powerful and precise actuation of the robot.

For this specific experiment, OneDOF was mounted on a custom made support in order to allow subjects to sit near to the robot (Figure 2B). Moreover a leg-shaped thermoplastic polymer was anchored to OneDOF in order to secure the subject's leg (Figure 2C).

#### 2.1.2. Data acquisition

**Figure 3:**
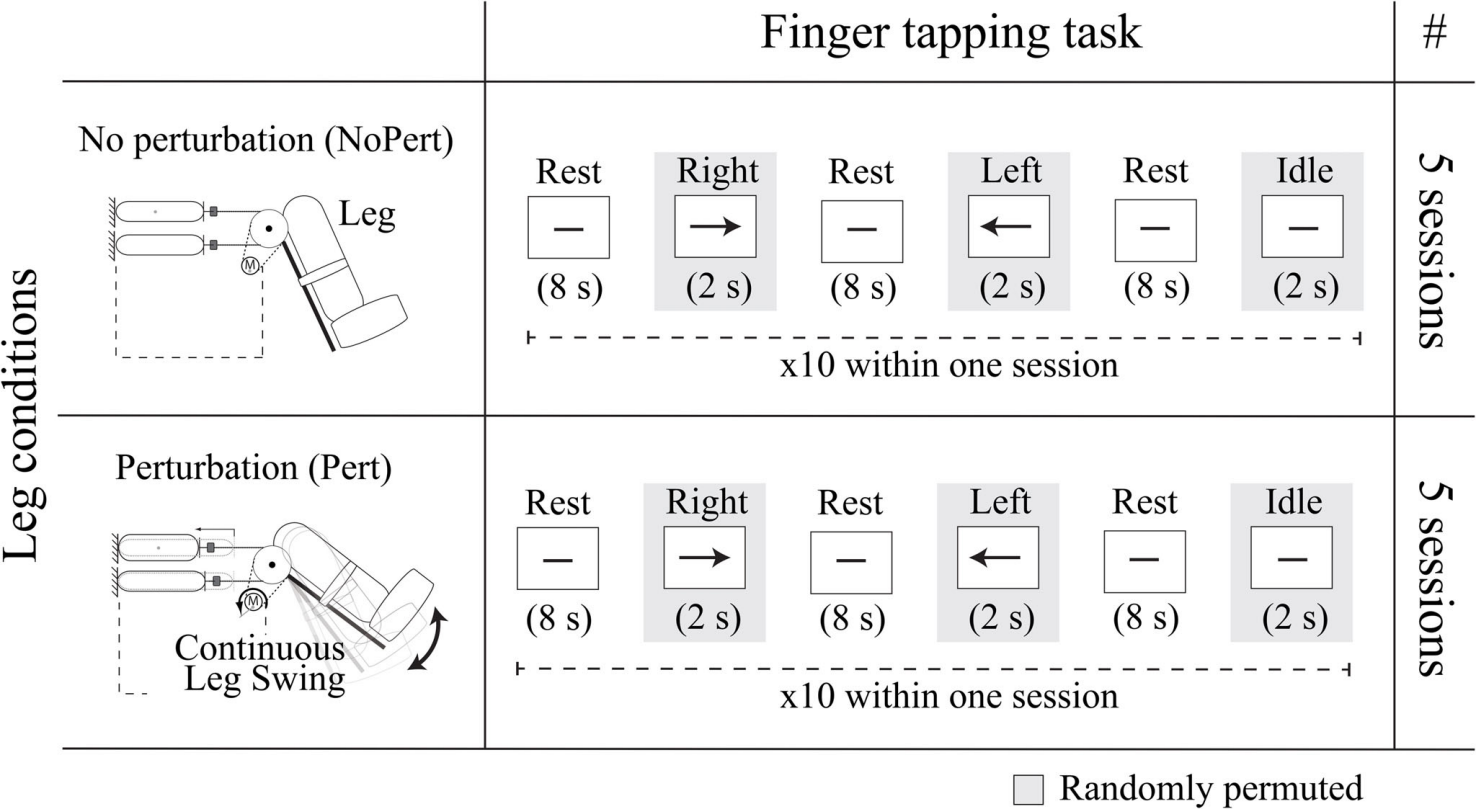
**Experimental design**. The experiment is structured as a 3 × 2 factorial design, consisting of two factors “finger tapping task” and “leg condition.” The former is divided into three levels, being left hand finger tapping (Left), right hand finger tapping (Right) and Idle, whose temporal representation is visualized in the “Finger tapping task” column. The “leg condition” factor is divided into two levels *NoPert* and *Pert*, which are represented, respectively, in the first and second rows of the table. To note that *Rest* is the resting period between tasks and that the visual cues of the tasks are randomly permuted within a session. Moreover, in the last column, the number of sessions performed for each experimental condition are visualized.

The experiment, which was carried out with five healthy subjects aged 23–27, can be represented by a 3 × 2 factorial design (Figure 3) consisting of two factors: “finger tapping task” and “leg condition.” The former is divided into three levels, being left tapping, right tapping and idle; while the latter is composed by two levels: *NoPert* and *Pert*.

With regard to the “leg condition” factor, five sessions of the experiment were performed while the assistive robot was stopped (*NoPert*) and other five while the robot continuously swung up and down at a frequency of 0.5 Hz between 5° and 50° of the knee joint (*Pert*).

During all the 10 sessions of the experiment the finger tapping task was carried out as follows: an hyphen was shown on the screen for 8 s to indicate that the subject should not move (rest). After this period, a left or right arrow randomly appeared, or the hyphen was maintained on the screen, for 2 s, indicating respectively that the subject should perform left or right hand brisk continuous finger tapping, or keep not moving (idle). Within one session, each of the three tasks, Left/Right tapping and Idle, was performed 10 times, resulting in 30 trials per session. After each session, subjects rested for about 5 min. Moreover, the EEG signal was recorded at a sampling rate of 2048 Hz with a 64-electrode cap and a Biosemi Active Two system for amplification and analog-to-digital conversion.

In order to confirm that to each visual cue corresponds a motor action, the finger tapping performance was visually observed by the experiment operator. Moreover, for the first three subjects the experiment was recorded on video, while for the other two subjects the index finger angle was measured by means of a goniometer sensor (Biometrics Ltd).

Subjects gave written informed consent for the experimental procedures, which were approved by the ATR Human Subject Review Committee.

### 2.2. Decoder training

In this section, we introduce the methodology to train and test the decoder that is used in the cross-validation procedure described in section 2.3. Moreover, we refer to “training set” and “test set,” detailed as well in section 2.3, but for the time being it is sufficient to think of them as two independent datasets that are used to, respectively, fit the parameters of a model (decoder) and evaluate the predictive power of the trained (fitted) model.

**Figure 4:**
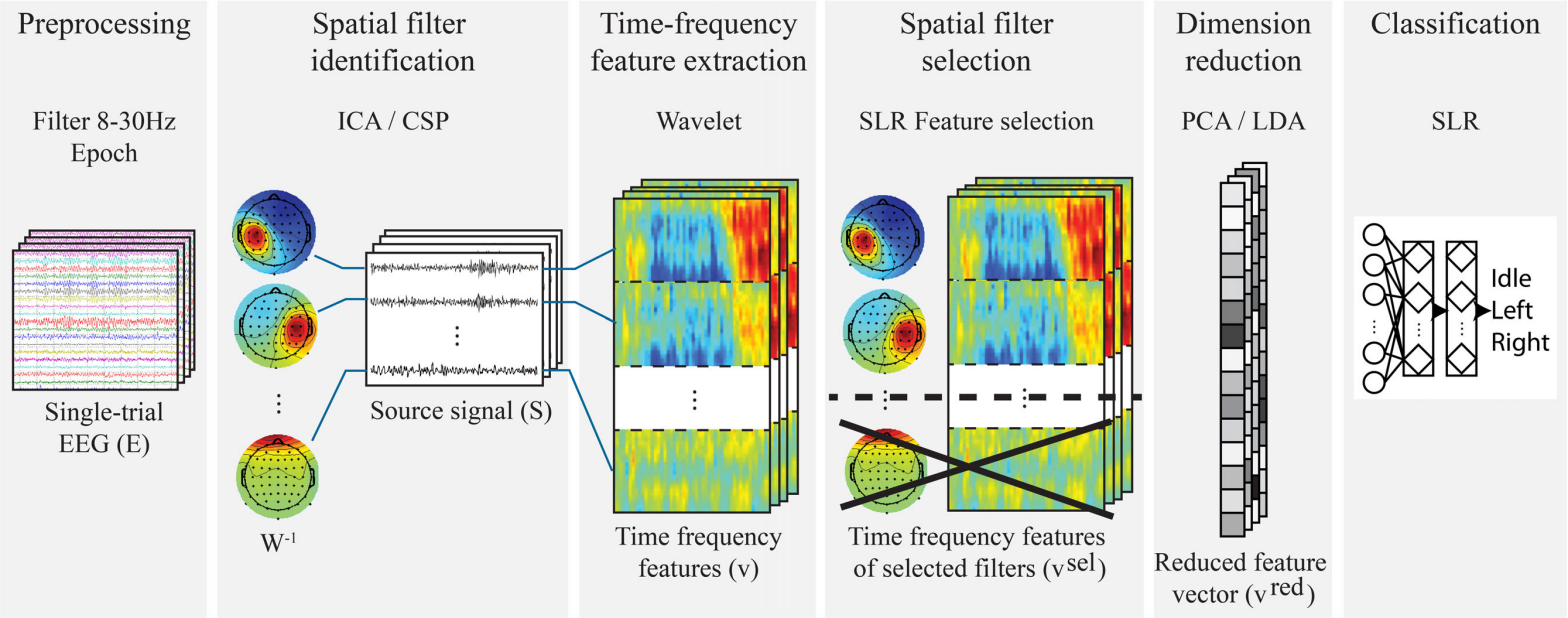
**Decoder training in a single fold of the cross-validation**. In the *Unsupervised feature extraction*-based version, ICA and PCA are used, respectively, for spatial filter selection and dimension reduction, while CSP and LDA are, respectively, employed in the *Supervised feature extraction*-based one. Important to note that the transformation matrices of spatial filter identification, spatial filter selection and dimensionality reduction are computed only on the training set of each cross-validation fold. Moreover, the time-frequency features are vectorized in the algorithm, while in figure they are kept in matrix form for a visualization purpose: the horizontal axis represents the time, and the vertical axis represents the frequency.

The six steps of the training algorithm (Figure 4) are: *1. preprocessing* (section 2.2.1), *2. spatial filter identification* (section 2.2.2), *3. wavelet transform for time-frequency feature extraction* (section 2.2.3), *4. spatial filter selection* (section 2.2.4), *5. feature dimensionality reduction* (section 2.2.5) and *6. training of a classifier* (section 2.2.6). In order to produce results that are independent from the characteristics of a specific feature extraction method, two types of decoder are used separately:

- *Unsupervised feature extraction*-based: the spatial filter identification step is performed by Independent Component Analysis (ICA) and feature dimensionality reduction by Principal Component Analysis (PCA);
- *Supervised feature extraction*-based: the spatial filter identification is carried out by Common Spatial Patterns (CSP) and feature dimensionality reduction by Linear Discriminant Analysis (LDA).

It is important to stress that the *Unsupervised feature extraction*-based decoder is trained and tested independently from the *Supervised feature extraction*-based one.

#### 2.2.1. Preprocessing

This step is common to both the *Unsupervised* and *Supervised feature extraction*-based decoders. Only a subset of 35 electrodes, centered at the motor cortex, is used: all the electrodes positioned within the ranges F5-F6, FC5-FC6, C5-C6, CP5-CP6, P5-P6. The training and test sets are resampled at 128 Hz and bandpass filtered (FIR filter implemented in EEGLAB, Delorme and Makeig, 2004) in the range from 8 to 30 Hz, encompassing the mu and beta frequency bands, which have been shown to be most important for movement classification. Epochs are extracted with respect to the visual cues presented to the subjects. In particular, the epoch starts 0.5 s before the cue onset and ends 2.5 s after the offset, for a total of 5 s. The time window after the movement offset is needed to capture completely the ERS, also considering that, sometimes, this is slightly delayed due to the subject's reaction time. In the following subsections **E**_*j*_ ∈ ℝ^*c* × *t*^ represents the single-trial EEG signals of the training set, where *c* is the number of channels, *t* is the number of time samples and *j* = 1 … n where *n* is the number of training trials.

#### 2.2.2 Spatial filter identification (ICA and CSP)

Previous studies have demonstrated that spatial filters are useful in single-trial analysis, in order to improve the signal-to-noise ratio (Blankertz et al., 2008b). For this purpose, Independent Component Analysis (ICA) is implemented in the *Unsupervised feature extraction*-based decoder, while Common Spatial Patterns (CSP) is used in the *Supervised feature extraction*-based one. Both ICA and CSP transform the observed EEG signals as:

(1) $$S_{j}=WE_{j}$$

where **E**_*j*_ represents the observed single-trial EEG signals, **W** the unmixing matrix, **S**_*j*_ the recovered single-trial sources and *j* = 1 … *n*, where *n* is the number of training trials.

***2.2.2.1. Independent Component Analysis (ICA)***. ICA is an unsupervised method that finds a linear transformation (*W*) of non-gaussian data (**E**), so that the resulting components (**S**) are as statistically independent as possible (Hyvärinen and Oja, 2000). Hence, the EEG signal is separated into Independent Components (ICs) accounting for different neural activities, but also stereotyped non-brain artifact signals including eye movements, line noise and muscle activities (Makeig et al., 2004). In this study, the logistic infomax ICA algorithm, implemented in the EEGLAB function *binica* (Delorme and Makeig, 2004), is executed on the preprocessed training set. This yields an unmixing matrix **W** ∈ ℝ^*s* × *c*^ and source signals (independent components) **S**_*j*_ ∈ ℝ^*s* × *t*^, where *s* is the number of sources, *c* the number of channels, *t* is the number of time samples and *j* = 1 … *n*, where *n* is the number of training trials.

***2.2.2.2. Common Spatial Patterns (CSP)***. CSP computes the unmixing matrix **W** to yield features whose variances are optimal for discriminating two classes of EEG measurements (Ramoser et al., 1998; Blankertz et al., 2008a) by solving the eigenvalue decomposition problem

(2) $$\Sigma_{1}W=(\Sigma_{1}+\Sigma_{2})WD$$

where **Σ**_1_ and **Σ**_2_ represent the estimates of the covariance matrices of the EEG signal associated with two different tasks, the diagonal matrix **D** contains the eigenvalues of **Σ**_1_ and the column vectors of **W**^−1^ are the filters for the CSP projections. The best contrast is provided by those filters with the highest and lowest eigenvalues, therefore the common practice is to retain *e* eigenvectors from both ends of the eigenvalue spectrum (Blankertz et al., 2008a). CSP is applied in a One-vs-Rest (OvR) fashion, in order to cope with the multi-class nature of the problem, separately (Dornhege et al., 2004) for three different frequency bands 8–13 Hz (*μ*), 15–25 Hz (*β*), 8–30 Hz (*μ* and *β*), in the time segment starting 1 s after the cue (Blankertz et al., 2008b). Moreover, *e* = 2 eigenvectors from the top and from the bottom of the eigenvalue spectrum are retained. This procedure is performed only on the preprocessed training dataset, yielding the unmixing matrix **W** ∈ ℝ^*s* × *c*^ and source signals **S**_*j*_ ∈ ℝ^*s* × *t*^, where *s* = 2 × *e* × 3 (frequency bands)× 3 (classes) is the number of sources (CSP projections), *c* is the number of channels, *t* is the number of time samples and *j* = 1 … *n*, where *n* is the number of training trials.

It is important to note that, with both ICA and CSP, the unmixing matrix **W** is computed *only using the training set*.

#### 2.2.3. Time-frequency analysis: the wavelet transform

In both the *Unsupervised* and *Supervised feature extraction*-based decoders, the Morlet Wavelet transform (Daubechies, 1990) is employed to extract time-frequency features, representing the subject-specific ERD/ERS patterns, from the source signals **S**. For each trial, a wavelet coefficient matrix with 50 time samples and 20 frequency bins is computed for the *i*-th source signal. The resulting coefficients are squared to get the spectral power and the 10log_10_ transformation is computed to obtain a final time-frequency representation (**c**_*i*_). Therefore, the feature vector of the *j*-th trial (**v**_*j*_) is obtained by the concatenation of the time-frequency coefficients **c**_*i*_ computed from the *i*-th source signal inside **S**_*j*_:

(3) $$v_{j}=\left[\begin{array}{c} \begin{array}{l} c_{1}\\ c_{i}\\ \vdots \\ c_{s} \end{array} \end{array}\right],c_{i}={\left[\begin{array}{c} c_{11},\cdots ,c_{f1},c_{12},\cdots ,c_{f2},\cdots ,c_{1t},\cdots ,c_{ft} \end{array}\right]}^{⊤}$$

In the equations above, **v**_*j*_ represents the *j*-th feature vector, *j* = 1 … *n*, where *n* is the number of training trials, **c**_*i*_ is the time-frequency coefficients vector of the *i*-th source, *i* = 1 … *s* where *s* is the number of sources in **S**_*j*_, *t* is the number of time samples and *f* is the number of frequency bins.

#### 2.2.4. Spatial filter selection based on SLR

Not all the spatial filters that have been previously identified by ICA or CSP are related to the neural processes of the tapping task. In particular, ICA identifies a large number of independent components that account for artifacts and other neural sources, while CSP might return some spatial patterns that over-fit the training set. Therefore, in order to avoid over-fitting and to obtain results that are not influenced by artifacts, it is necessary to reduce the number of spatial filters to the ones that are strictly indispensable for the classification of the tapping task.

For this purpose, Sparse Logistic Regression (SLR) is used to select the most important spatial filters based on their time-frequency features. SLR is a Bayesian extension of logistic regression, which simultaneously performs feature selection and training of model parameters for classification. It utilizes automatic relevance determination (ARD) to determine the importance of each parameter while estimating its values. This process selects only a few parameters as important and prunes away others. The resulting model has a sparse representation with a small number of estimated parameters (Yamashita et al., 2008). In this work we apply the OvR version of the algorithm to cope with the multi-class problem. Moreover, prior to training, each feature of the training set is normalized using its mean across trials and scaled using the respective standard deviation. Furthermore, the mean and standard deviation of the training features are used to normalize also the feature vectors of the test set.

SLR can be used to assign scores to spatial filters based on the classification performance and selection recurrence of their time-frequency features. In the current study we were inspired by the method proposed by Yamashita et al. (2008), which selects features on the basis of their selection recurrence. Given the fact that our goal is not to select single features, but spatial filters which are, in turn, represented by a set of time-frequency features we had to modify the original algorithm. The revised method considers the recurrence of the group of time-frequency features belonging to each spatial filter. The basic idea is that spatial filters whose time-frequency features are repeatedly selected with good classification performance among a variety of training data sets could be important.

Specifically, the selection of spatial filters is implemented by estimating SLR weight parameters on 80% of the *training set* and evaluating the classification performance on the remaining 20% of the *training set*. This process is *repeated five times* so that each trial of the training set is used once to evaluate the performance. Therefore, we can define a score value for each *i*-th spatial filter (*i* = 1 … *s* where *s* is the number of sources in **S**_*j*_) based on the number of selected features that belong to it. More precisely, let *θ*_*j*_ and *p*_*j*_ denote the estimated parameter vector and classification performance (percent) resulting from the *j*-th repetition of SLR (*j* = 1 … *R*, where *R* is the number of SLR repetitions). Moreover, *θ*_*j*_(*k*) = 0 if the *k*-th feature is not selected (*k* = 1 … |*θ*| where |*θ*| is the length of the feature vector). Then the score value (SC) for the *i*-th spatial filter (SF) is defined by:

(4) $$SC_{i}=\sum_{j}^{R}\sum_{k\in SF_{i}}^{\left|\bar{\theta }\right|}I({\bar{\theta }}_{j}(k)\neq 0)\times p_{j}$$

where *I*( · ) denotes an indicator function that takes the value of 1 if the condition inside the brackets is satisfied, 0 otherwise. *R* = 5 is the number of SLR repetitions, |*θ*| is the length of the feature vector and *θ*_*j*_(*k*) is the estimate of the *k*-th parameter belonging to the *i*-th spatial filter (SF), with respect to the *j*-th repetition. Once SC has been computed for all the spatial filters, we retain only those whose SC is above a given threshold. Specifically for selecting independent components the threshold is *μ*_*SC*_ + *σ*_*SC*_, while for CSP the threshold is simply *μ*_*SC*_, where *μ* and *σ* represent respectively mean and standard deviation of the SC values. The reason for having two different thresholds is that ICA returns a large number of independent components, not related to the classification, that need to be removed, such as artifacts and other neural sources. On the other hand, being CSP a supervised method, it selects mainly spatial filters that are important for the classification, and we only remove the few ones that would cause over-fitting.

Supposing that the set *D* of selected filters contains *d* < *s* components (*s* total number of sources in **S**_*j*_), the new feature vector **v**^*sel*^_*j*_ is obtained by concatenating the time-frequency features of the selected filters **c**_*i* ∈ *D*_:

(5) $$v_{j}^{sel}=\left[\begin{array}{c} \begin{array}{l} c_{1}\\ c_{i}\\ \vdots \\ c_{d} \end{array} \end{array}\right],c_{i\in D}={\left[\begin{array}{c} c_{11},\cdots ,c_{f1},c_{12},\cdots ,c_{f2},\cdots ,c_{1t},\cdots ,c_{ft} \end{array}\right]}^{⊤}$$

In the equations above, **v**^*sel*^_*j*_ represents the feature vector associated with the selected spatial filters of the *j*-th trial, **c**_*i* ∈ *D*_ the time-frequency coefficient vector of the *i*-th source belonging to the set of selected filters *D, j* = 1 … *n* where *n* is the number of training trials, *i* = 1 … *d* where *d* is the cardinality of *D, t* is the number of time samples and *f* is the number of frequency bins. Therefore, the *j*-th feature vector **v**^*sel*^_*j*_ is composed by *l* = *d* × *f* × *t* elements. Moreover, the spatial filter unmixing matrix takes the following form **W**^*sel*^ ∈ ℝ^*d* × *c*^, where *c* is the number of channels and *d* the number of selected spatial filters. To note that the SLR-based spatial filter selection is performed *only on the training set*.

#### 2.2.5. Dimensionality reduction (PCA and LDA)

Even though the spatial filter selection method reduces the number of sources, the dimensionality of the concatenated time-frequency feature vector is in the order of thousands elements. Moreover, *the time-frequency features are somewhat redundant, because the values of adjacent points in the spectrogram are highly correlated*. Therefore, it is important to perform dimensionality reduction. For this purpose, Principal Component Analysis (PCA) is employed in the *Unsupervised feature extraction*-based decoder, while Linear Discriminant Analysis (LDA) in the *Supervised feature extraction*-based one.

PCA and LDA are, respectively, the most popular unsupervised and supervised dimensionality reduction methods in literature (Wang and Paliwal, 2003) and they both reduce the features dimension by projecting the original feature vector into a new feature space through a linear transformation matrix. Nonetheless, they optimize the transformation matrix with different intentions: PCA optimizes the transformation matrix by finding the largest variations in the original unlabeled feature space, while LDA pursues the largest ratio of between-class variation and within-class variation when projecting the original labeled features to a subspace (Wang and Paliwal, 2003). In this study PCA is implemented by Singular Value Decomposition (SVD), while LDA is executed by means of the algorithm proposed by Cai et al. (2008). To note that prior to performing PCA or LDA the mean of the training set is subtracted, and that in the case of PCA the mean of the training set is also subtracted from the test set.

The dimensionality reduction step yields a linear transformation matrix **A** ∈ ℝ^*p* × *l*^ that projects the original feature vector **v**^*sel*^_*j*_ ∈ ℝ^*l*^ to the reduced vector **v**^*red*^_*j*_ ∈ ℝ^*p*^, where *l* is the length of the original feature vector and *p* the number of projections (reduced features, *p* < *l*). When dimensionality reduction is performed by PCA, the number of retained principal components (*p*) is set so as to achieve 90% of variance (Jolliffe, 2002), bringing the length of the feature vector from the order of thousands to the order of hundreds elements. On the other hand, when using LDA, the number of projections *p* = 2 since the number of non-zero eigenvalues is bounded by *g* − 1, where *g* is the number of classes (Cai et al., 2008). Once again, it is important to note that, with both PCA and LDA, the projection matrix *A* is computed only using the training set.

#### 2.2.6. Classification

Once the feature vectors of all the trials in the training dataset (**v**^*red*^_*j*_) are obtained, it is possible to compute the ones of the test dataset ($\hat{v}$^*red*^_*k*_), by the following steps:

1. to generate the source signal matrix of the *k*-th trial in the test set (**S**^*test*^_*k*_), multiply the preprocessed *k*-th single-trial EEG of the *test dataset* (*k* = 1 … *m*, where *m* is the number of test trials) by the spatial filter unmixing matrix **W**^*sel*^, obtained on the training set by *spatial filter selection* (section 2.2.4);
2. to generate the test feature vectors $\hat{v}$^*sel*^_*k*_, compute the time-frequency matrix for each trial *k* and source in **S**^*test*^_*k*_;
3. to generate the reduced feature vector $\hat{v}$^*red*^_*k*_, multiply $\hat{v}$^*sel*^_*k*_ by the projection matrix **A**, obtained on the training set by *dimensionality reduction* (section 2.2.5).

The reduced feature vectors of the training set **v**^*red*^_*j*_ and the one of the test set $\hat{v}$^*red*^_*k*_ are used, with the respective labels, to train and evaluate a SLR classifier (introduced in section 2.2.4). To note that the SLR classifier used to decode the finger tapping task is trained and tested on features and datasets that differ from to the ones used to select spatial filters in section 2.2.4.

### 2.3. Performance assessment

The main objective of this study is to verify that the decoding performance of the upper limb tasks is not affected by the lower limb periodic perturbation. To do so, we compare the performance of the finger tapping decoding under the two conditions *NoPert* and *Pert*. In other words, from a factorial point of view, the goal is to assess the main effect of leg perturbation on the decoding of finger tapping. Given the fact that the leg perturbation might influence each subject's performance in a different way, an intra-subject analysis is carried out.

Individually for each subject, the datasets of 9 out of 10 sessions are used as “training set,” to train the decoder described in section 2.2, and the dataset of the remaining session is used as “test set.” This procedure is repeated 10 times so as to use each session as test set once and only once, where a repetition is named “fold” and the whole operation is called 10-fold cross validation. Therefore, 5 out of 10 folds are characterized by a test set which belongs to the *NoPert* condition and the remaining 5 folds are characterized by a test set belonging to the *Pert* condition. In this way, the decoding performance of the test sets belonging to the *NoPert* condition is computed separately from the one of the *Pert* condition, in order to carry out a statistical comparison of the two.

Specifically, performance is evaluated by Cohen's Kappa (Cohen, 1960), also employed in the BCI Competitions (Tangermann et al., 2012):

(6) $$k=\frac{Pr(a)-Pr(e)}{1-Pr(e)}$$

where *Pr*(*a*) is the proportion of observed agreements, and *Pr*(*e*) is the proportion of agreements expected by chance. The range of possible values of Kappa is between −1 and 1, though it usually falls between 0 and 1. Perfect agreement between the true target labels and the predicted ones is represented by *k* = 1. Agreement no better than that expected by chance is indicated by *k* = 0. A negative kappa would indicate agreement worse than that expected by chance (Sim and Wright, 2005). An advantage of using the Kappa coefficient is the possibility to perform a *Z*-test to compare two classifications and determine if the accuracy level between the two is significantly different (Congalton et al., 1983, see Appendix for details).

## 3. Results

### 3.1. Decoding performance

Table 1 represent the results obtained, respectively, by the *Unsupervised feature extraction*-based decoder and by the *Supervised feature extraction*-based one. The table contain the Kappa values for each subject, relative to the conditions *NoPert* and *Pert*, and the associated intra-subject comparison (*Z*-score). We observe that all the Kappa scores are above chance level (*K* = 0). Importantly, the comparisons between *NoPert* and *Pert* has always a *Z*-score <1.96, therefore, for every subject, the two conditions are not significantly different from a classification performance point of view.

**Table 1:** **Intra-subject comparison of Kappa associated with *NoPert* and *Pert*, and the relative *Z*-score**.

**a. Unsupervised feature extraction**				**b. Supervised feature extraction**			
**Sbj**	**K(NoPert)**	**K(Pert)**	**Z-score**	**Sbj**	**K(NoPert)**	**K(Pert)**	**Z-score**
S1	0.95	0.81	1.47	S1	0.97	0.85	1.23
S2	0.85	0.75	1.24	S2	0.79	0.67	1.47
S3	0.59	0.58	0.02	S3	0.58	0.48	1.16
S4	0.79	0.64	1.75	S4	0.63	0.67	0.42
S5	0.68	0.75	0.90	S5	0.76	0.76	0.02

Two conditions are significantly different (α = 0.05) if Z-score is >1.96. The table shows the results of the Unsupervised and Supervised feature extraction-based decoders. To note that chance level is K = 0.

### 3.2. Feature visualization

In order to interpret the results, the features used in the decoding process are visualized separately for each subject. Specifically, we focus on the features of the *Unsupervised feature extraction*-based decoder, since ICA yields unmixing matrices (**W**) that are similar along cross-validation folds. This means that ICA components can be clustered along folds, and for each cluster, topographies and time-frequency patterns can be averaged. Nonetheless, CSP patterns differ along cross-validation folds, given the supervised nature of CSP and the fact that the training set varies from fold to fold. Therefore, it is not possible to cluster and average the CSP features. For these reasons we decided to focus on the feature visualization of the *Unsupervised feature extraction*-method. Nonetheless, we report that, for each fold and subject, CSP filters and the associated ERD/ERS activations are compatible with the ones related to the *Unsupervised feature extraction* method.

#### 3.2.1. Topographic maps

**Figure 5:**
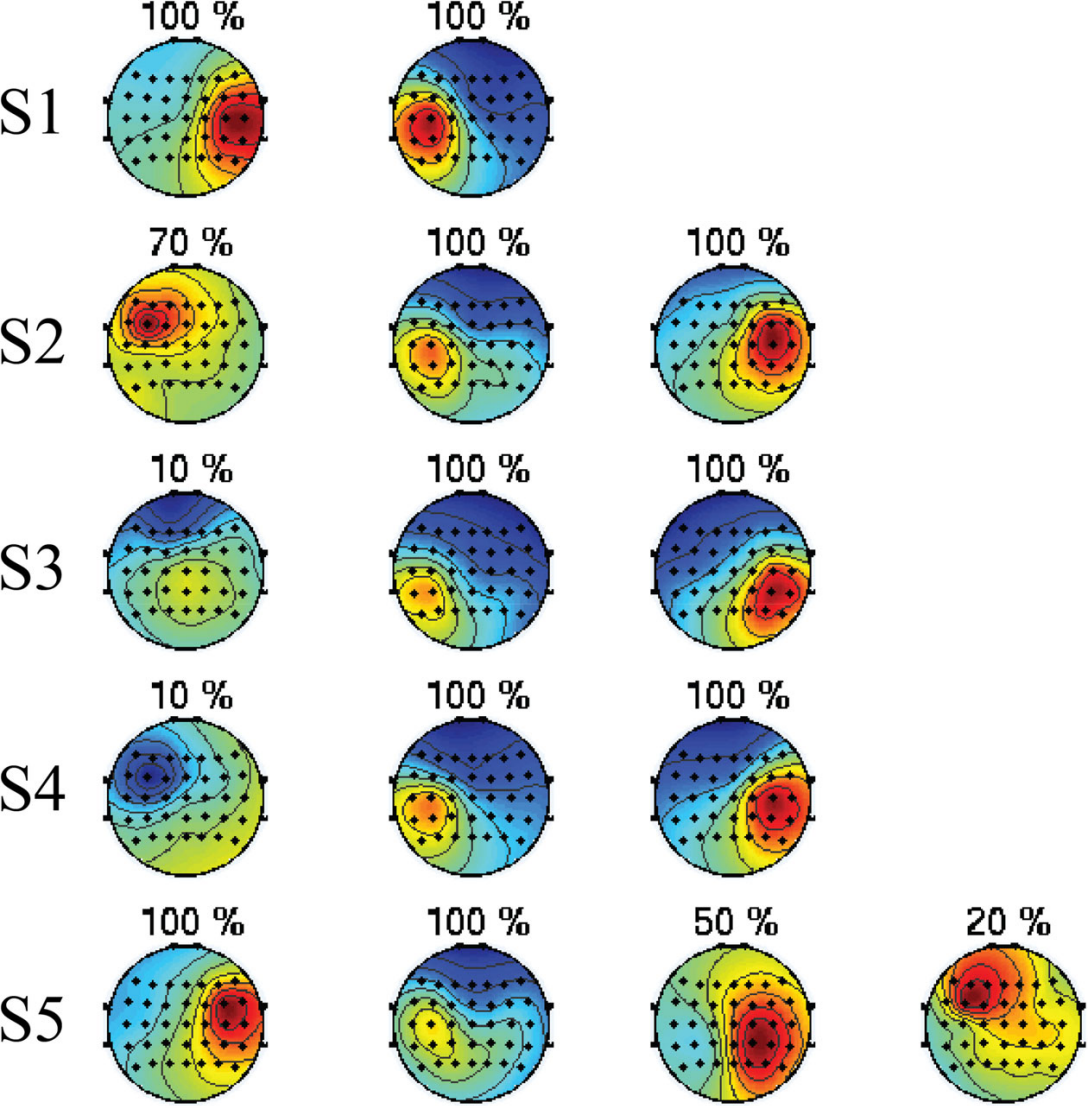
**Topographies of the automatically selected ICA components**. Each row represents one subject, and each topographic map represents a cluster of ICA components obtained by hierarchical clustering along cross-validation folds. On top of each map there is the selection ratio *r* representing how frequently a member of the cluster is selected along folds. Spatial filters with a selection ratio *r* ≥ 50% represent, for every subject, either the activation of the left or right hemisphere. Moreover, it is important to note that the selected spatial filters cover areas of the EEG electrode space that are different from subject to subject.

The spatial filters extracted after ICA and SLR spatial filter selection (**W**^*sel*^) are visualized in this section. In order to obtain a compact representation, they are clustered across folds, separately for each subject. Specifically, the rows of **W**^*sel*^ are clustered by means of hierarchical clustering, in such a way that only rows with a small Euclidean distance are grouped together. Moreover, for each cluster *C* we compute the selection ratio, representing how frequently a member of the cluster is selected along cross-validation folds: *r* = |*C*|/*k*, where |*C*| represents the cardinality of C and *k* is the total number of cross-validation folds. In Figure 5 the topographic maps of the average of each cluster and the respective selection ratio *r* are displayed separately for every subject. The figure shows that spatial filters with a selection ratio *r* ≥ 50% represent, for every subject, either the activation of the left or right hemisphere. Moreover, it is important to note that the selected spatial filters cover areas of the EEG electrode space that are different from subject to subject.

#### 3.2.2. Time-frequency features

In order to visualize the time-frequency features of the *Unsupervised feature extraction*-based decoder, the event-related spectral perturbation (ERSP, Grandchamp and Delorme, 2011) is employed:

(7) $$\begin{array}{l} ERSP(f,t)=10log_{10}\left(\frac{ERS(f,t)}{\mu_{B}(f)}\right),\\ \;ERS(f,t)=\frac{1}{n}\sum_{k=1}^{n}{\left|F_{k}(f,t)\right|}^{2} \end{array}$$

where *ERS* is the event related spectrum and is obtained by averaging across trials the time-frequency power estimate computed by wavelet (*F*), *μ*_*B*_(*f*) is the mean spectral estimate at baseline, *n* is the total number of trials, *F*_*k*_(*f, t*) is the spectral estimate at frequency *f* and time point *t* for trial *k*.

**Figure 6:**
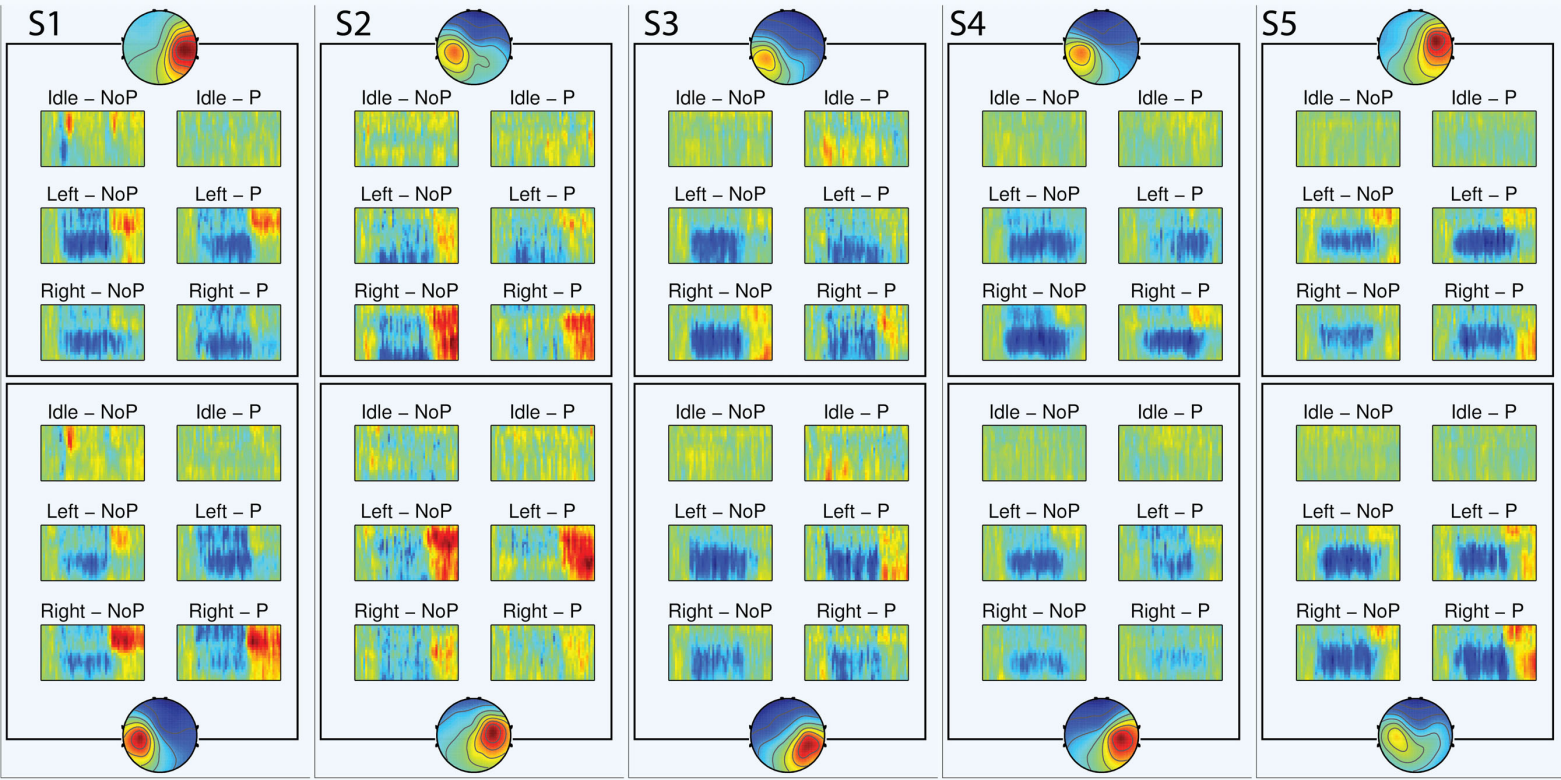
**ERSP visualization**. In each ERSP image, the abscissa represents the time (−0.3 to +4.3 s), while the ordinate represents the frequency (from 8 to 30 Hz), the red color portrays power increase with respect to the baseline (dB), while the blue color power decrease (dB). For each subject, only the ERSP of the spatial filters with a selection ratio *r* = 100% are visualized. The figure is organized so that it is possible to compare the ERSP of *NoPert* (NoP) and *Pert* (P), while appreciating the features that characterize each class. To note that the ERD/ERS patterns are subjects-specific and informative for the classification. Moreover this representation do not highlight differences between the two conditions *NoPert* and *Pert*, confirming the quantitative results of the *Z*-test.

The ERSP is computed separately for the test datasets of *NoPert* and *Pert*, for each subject and class (Idle, Left trapping, Right tapping). Thus, Figure 6 is organized so that it is possible to compare the spectral perturbation associated with the two conditions (*NoPert, Pert*), while appreciating the features that characterize each class, individually for each subject. To note that only the ERSP of the spatial filters with a selection ratio *r* = 100%, discussed in the previous section, are visualized. The ERSP visualization does not highlight differences in the ERD/ERS pattern between *NoPert* and *Pert* conditions. Moreover, we observe that the spectral perturbations are actually informative for the classification. In particular, the ERSP of the *Idle* class does not contain ERD/ERS, while the remaining two classes are characterized by strong spectral perturbations. Furthermore, we note subject-specific differences in the ERSP of the *Left* and *Right* classes as well. Subject 1 (S1) is characterized by a stronger beta ERS of the right hemisphere when left tapping is performed and by beta ERS of the left hemisphere in case of right tapping (contralateral activation); S2 displays a similar class-specific contralateral mu and beta ERS; for S3 we observe a stronger mu ERD of the left hemisphere when left tapping is executed (ipsilateral activation), compared to the mu ERD of the right hemisphere associated with the right tapping; for S4 we observe a stronger contralateral mu ERD and, lastly, for S5 we note a stronger contralateral beta ERS.

## 4. Discussion

The aim of this study is to investigate the main effect of the leg afferent input, induced by a lower limb assistive robot, on the decoding performance of the sensorimotor hand area ERD/ERS. To do so, we devised an experiment to compare the finger tapping decoding performance under the conditions of leg perturbation (*Pert*) and no perturbation (*NoPert*). From the experimental results we find that the classification performance is always above chance (*K* > 0) and we do not observe intra-subject significant difference (*Z*-score <1.96, *p*-value >0.05) between the conditions *NoPert* and *Pert*.

To note that, in each cross-validation fold, a decoder is trained on both conditions. This is motivated by the fact that it is important to evaluate the performance on a common model. Training and testing a decoder separately for *NoPert* and *Pert* wouldn't allow for a sound comparison, since the underlying models would be different. Moreover, the datasets representing the two conditions might differ due to non-stationarities that are not related to the leg perturbations (e.g., electrode impedance variation, Wojcikiewicz et al., 2011). Training across both conditions should minimize the effect of those non-stationarities, while testing separately between *NoPert* and *Pert* should highlight the actual differences caused by leg perturbations.

In order to corroborate the interpretation of the quantitative analysis carried out so far, we visualize only the features of the *Unsupervised feature extraction*-based decoder in Figures 5, 6. This is motivated by the fact that spatial filters obtained by ICA are more stable along cross-validation folds. Therefore, they can be clustered and the ERSP of each cluster can be computed in a meaningful way. From this analysis we confirm that the spatial patterns with high selection ratio (*r* ≥ 50%) are compatible with the spatial mapping of the sensorimotor hand area ERD/ERS suggested by (Pfurtscheller and Lopes da Silva, 1999). Moreover, the fact that spatial topographies are different from subject to subject is in line with the thesis of Blankertz et al. (2008b), asserting that subject-specific spatial filters are important to enhance the decoding performance. Not only spatial patterns, but also the ERD/ERS patterns are highly subject-specific (Pfurtscheller and Neuper, 2006): compatibly with Pfurtscheller and Lopes da Silva (1999), for some subjects the discriminability between the three classes is to be inputed to strong post movement mu and/or beta ERS (S1, S2, and S5), while for other subjects we observe a predominance of mu ERD (S3 and S4). Importantly, the ERSP visualization does not highlight differences in the ERD/ERS pattern between *NoPert* and *Pert* conditions, which confirms the quantitative results of the *Z*-test.

One possible explanation to the fact that the ERD/ERS patterns associated with upper limb movements are not significantly changed by lower limb afferent input is that periodic lower limb passive movements do not produce EEG perturbation that are as significant as the ones of active movements. This is in line with other studies (Wagner et al., 2012; Jain et al., 2013) reporting a significant mu and beta desynchronization, at the primary motor cortex, associated with active periodic movements as opposed to passive periodic movements of the lower limbs. Nonetheless, in Müller-Putz et al. (2007) it is observed that for *non-periodic* passive and active movements, the beta ERD/ERS have similar levels of significance at the central sensorimotor and surrounding areas (electrodes Cz, C2, and Fcz). Therefore, it is possible that in the current study, being the leg perturbations periodic, they did not elicit ERD/ERS significant enough to interfere with the EEG perturbations associated with the upper limb task.

This paper focuses on one of the many aspects regarding the implementation of a brain-controlled assistive robot. Especially, it is important to highlight that, even though EEG topographies and ERD/ERS patterns are shared between actual movements and motor imagery (Pfurtscheller and Lopes da Silva, 1999; McFarland et al., 2000), the latter is characterized by a significantly smaller ERD/ERS magnitude (McFarland et al., 2000; Solis-Escalante et al., 2010). This raises the question, whether the EEG decoding of a motor imagery task might be affected by the leg afferent input. This issue will be investigated in future studies. Nonetheless, we need to stress the necessity of the current study, which establishes a “ground truth” understanding of the possible interference between the ERD/ERS of the sensorimotor area and the lower limb afferent input. In this context, the term “ground truth” is related to the fact that actual movements are observable by the experiment operator, or mechanically measurable, therefore we are certain that a motor command is elicited for every cue presented to the subject. On the other hand, motor imagery cannot be visually observed by the experiment operator, therefore, in case of misclassification it is impossible to say whether the subject failed to elicit motor imagery ERD/ERS or the leg perturbation actually inhibited it.

Moreover, it is important to note that finger movements are characterized by a significantly smaller EEG power perturbation as compared to hand movements (Pfurtscheller et al., 1998). This means that the finger tapping task is a good compromise in order to keep the spectral perturbation magnitude as close as possible to the one of motor imagery, while preserving the necessary property of mechanical measurability, or observability by the experiment operator.

Another issue, that must be addressed in future studies, regards the influence of a multi-DoF assistive robot on the ERD/ERS decoding. Especially, it remains an open question how the afferent input, induced by the passive movement of the whole lower limbs, affects the EEG decoding. An fMRI study by Newton et al. (2008) found a substantial overlap of the motor representations of ankle dorsiflexion, ankle plantarflexion and knee extension. This supports the notion that the afferent input induced by a multi-DoF robot should not differ significantly from the one induced by OneDOF robot. Nonetheless, further investigations are required to clarify this point.

Additionally, it is of interest whether reflex responses of the major leg muscles could influence the EEG patterns. For this purpose, in future experiments the electromyographic (EMG) signal will be recorded from the lower limbs. In this way, it would be possible to detect the onsets of possible reflex muscle activations at the leg, and further analyze the EEG signal based on this information.

Moreover, another concern regarding the application of our approach to a multi-DoF assistive robot is represented by the mechanical artifacts affecting the EEG signal. However, other researchers have shown that artifacts during walking (Gwin et al., 2010) and robotic-assisted treadmill walking (Wagner et al., 2012) can be significantly reduced by means of ICA, encouraging further studies with the multi-DoF assistive robot.

In conclusion, this study does not find a main effect of the leg afferent input, induced by a lower limb assistive robot, on the decoding performance of the sensorimotor area ERD/ERS. This establishes a solid ground for future experiments and studies aimed at controlling a multi-DoF assistive robot by motor imagery. A possible future application of such a Brain Robot Interface (BRI) system would be to modulate, by left or right motor imagery, the walking speed of an exoskeleton robot once it started moving.

### Conflict of interest statement

The authors declare that the research was conducted in the absence of any commercial or financial relationships that could be construed as a potential conflict of interest.

## A. Appendix

### A.1. *Z*-test

Being *k*_*N*_ and *k*_*P*_ the Kappa scores, respectively, of the *NoPert* and *Pert* conditions, the *Z*-score of their difference can be computed as:

(A1) $$z_{NP}=\frac{\left|k_{N}-k_{P}\right|}{\sqrt{var(k_{N})+var(k_{P})}}$$

where *var*(*k*), the variance of *k*, can be computed according to the original formula in Congalton et al. (1983). Thus, two Kappa values are considered significantly different, with a significance level of *p*-value <0.05 if their *Z*-score is bigger than 1.96.
